# Hydrogen Isotope and Water Content Analysis of Ultralow Volume Volcanic Glass

**DOI:** 10.1021/acsmeasuresciau.6c00092

**Published:** 2026-07-04

**Authors:** Zhiqiang Yu, Jialong Hao

**Affiliations:** Key Laboratory of Planetary Science and Frontier Technology, 66451 Institute of Geology and Geophysics, Chinese Academy of Sciences, Beijing 100029, China

**Keywords:** Hydrogen isotopes, H_2_O content, NanoSIMS, volcanic glass, Early Cretaceous

## Abstract

Hydrogen isotope and H_2_O content of hydrated volcanic glass are useful proxies for paleoelevation and paleoclimate reconstruction. However, their analysis remains challenging because conventional bulk methods typically require relatively large amounts (∼10^–3^–10^–2^ g) of purified and fresh volcanic glass. Volcanic glass from the Mesozoic sediments and scientific drilling core samples is commonly affected by alteration and grain-scale heterogeneity, and the available sample mass is often insufficient for bulk analysis. Here, we present an ultralow sample consumption (∼10^–9^ g) in situ method for determining H_2_O contents and hydrogen isotope of hydrated volcanic glass from Early Cretaceous tuffs. This approach extends such analyses to older and less-well-preserved materials and provides a practical basis for high-resolution paleoenvironmental reconstruction.

## Graphical abstract

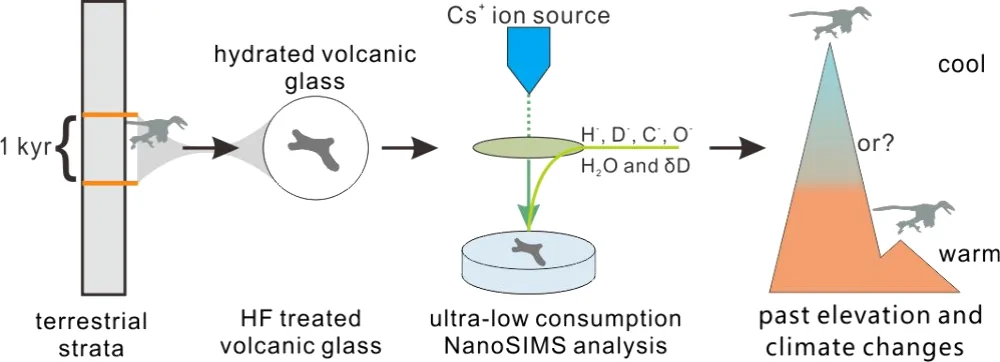

## Introduction

Hydrogen isotope and H_2_O contents of volcanic glass provide useful constraints on planetary volatile evolution, magmatic processes, and paleoenvironmental conditions recorded by hydrated glass.^1–7^ In particular, hydrated volcanic glass is a valuable record of paleowater and has been widely used to reconstruct paleoclimate and paleoelevation through time.

After deposition, hydration of volcanic glass begins to hydrate upon contact with environmental water and continues for 1–10 kyr (kyr = 10^3^ yr) until saturation is reached, a time scale that is rapid relative to most Cenozoic and pre-Cenozoic climate changes.^8,9^ During this process, the glass undergoes corrosion and alteration because of its amorphous, metastable nature, forming a thin, microporous, silica-rich layer near the glass surface.^10^ This layer substantially inhibits further exchange between environmental water and the glass interior, thereby limiting subsequent hydration and isotopic re-equilibration and favoring preservation of the hydration acquired hydrogen isotopic signature over geologic time scales.

Because the stable isotope composition of precipitation generally decreases with increasing elevation as a result of Rayleigh distillation during orographic uplift, the hydrogen and oxygen isotope values recorded by hydrated volcanic glass can provide insights into past topography and climatic evolution.^11^ Combined with the widespread occurrence of tuffaceous deposits, the common presence of dateable phenocrysts, and the potential for regional tephrostratigraphic correlation, hydrated volcanic glass constitutes a powerful archive for paleoenvironmental reconstruction with high temporal and spatial resolution. Despite this utility, applications of this paleoelevation and paleoclimate proxy have focused predominantly on Cenozoic deposits in North and South America (∼50–1 Ma, where Ma = 10^6^ yr),^11–13^ whereas its applicability to older volcanic glass (>100 Ma) remains poorly explored.

Currently, the main analytical approaches for determining H_2_O content and hydrogen isotope are thermal conversion elemental analyzer (TC/EA) and Fourier-transform infrared spectroscopy (FTIR). Among these, the application of TC/EA is limited by the relatively large sample mass (∼10^–3^–10^–2^ g) required, by contrast, FTIR is widely used for H_2_O content determination but cannot provide hydrogen isotope data. Recent work on valuable legacy scientific drilling cores^14,15^ and pre-Cenozoic ash materials has underscored an additional limitation of conventional bulk methods: many archived samples contain too little fresh volcanic glass for analysis. This constraint is especially severe in older, particularly Mesozoic, strata and in other low-volume materials such as scientific drilling core samples. In-situ analysis, particularly NanoSIMS, with its low sample consumption and the highest spatial resolution, holds promise for overcoming the aforementioned limitations and has already been applied to the determination of hydrogen isotope and water content in valuable planetary samples.^4,16^

Here we introduce a NanoSIMS strategy optimized for sample-limited volcanic glass and apply this protocol to older Early Cretaceous (∼125 Ma) volcanic glass. Requiring only nanogram-scale material, this method directly targets carefully screened fresh glass microdomains and avoids the inherent homogenization of bulk analyses. Our results highlight its strong potential for high-resolution paleoenvironmental reconstruction.

## Samples and Experimental Section

### Geological Background and Volcanic Glass Samples

The terrestrial rift basins in the North China Craton contain a nearly complete sedimentary succession of Lower Cretaceous strata.^17^ The fossils of the Jehol Biota occur within the Lower Cretaceous Huajiying Formation,^18^ Yixian Formation and Jiufotang Formation. In this study, we focus on the Lower Cretaceous Yixian Formation (∼125 Ma) in western Liaoning Province, northeastern China, which preserves remarkable fossil assemblages that have revolutionized our understanding of the Mesozoic vertebrate evolution and Early Cretaceous ecosystems.^19^

Two volcanic glass-bearing tuffaceous samples were collected from the Huangbanjigou No. 1 core,^20,21^ which was drilled directly adjacent to quarries yielding typical Jehol Biota fossil assemblages. The thickness of the Yixian Formation in this drill core is ∼ 9,130 cm. Tuff samples HBJ25–05 and HBJ25–03, which are 0.5 and 1 cm thick, respectively, were collected from horizons at core depths of 3,962 cm and 3,838 cm (Figure S1).

### Separation and Pretreatment of the Volcanic Glass

To obtain pure, fresh volcanic glass, we optimized a workflow including separation, hand-picking, HF pretreatment, and target preparation following published volcanic-glass extraction and purification procedures.^8,22^ Because the target glass grains are commonly associated with alteration phases and nonglass detritus, separated grains were first hand-picked and then briefly etched with 5% HF at room temperature for 40–50 s to remove unstable surface material. The residues were subsequently rinsed repeatedly with distilled water, washed thoroughly with ultrapure water, and dried prior to mounting.

Volcanic glass samples were mounted on Sn–Bi alloy targets together with San Carlos olivine (SCOl) as a hydrogen background monitor, and dried and vacuum-conditioned before analysis.^23^ Volcanic glass samples were further screened by reflected-light microscopy and backscattered electron (BSE) imaging to distinguish fresh glass from altered glass, feldspar fragments, and other nonglass phases. Only petrographically fresh and compositionally appropriate internal domains were ultimately selected for hydrogen isotope and H_2_O content analyses. The solidified Sn–Bi alloy mounts were ground using diamond sandpaper and polished with diamond suspension. Prior to hydrogen isotope and H_2_O content analyses, the mount is vacuum-coated with high-purity gold (99.999%), and a gold film of about 30 nm thickness is coated on the surface and side of the mount.

### Hydrogen Isotope and H_2_O Content Analysis of Volcanic Glass

Hydrogen isotope and H_2_O content measurements of volcanic glass were carried out on a NanoSIMS 50L instrument at the Institute of Geology and Geophysics, Chinese Academy of Sciences. H_2_O contents and hydrogen isotope were determined in situ on preselected fresh domains of individual shards. Under these conditions, the NanoSIMS chamber routinely reached vacuum levels as low as 1.4 × 10^–10^ mbar.

A Cs^+^ primary ion beam (350 pA, ∼ 400 nm spot size) was used in multicollector mode. Secondary ions of ^1^H^–^, ^2^D^–^, ^12^C^–^, and ^18^O^–^ were simultaneously detected using four electron multipliers. An entrance slit (ES-3) was employed, yielding a mass-resolving power (MRP) of ∼ 4500 (10–90% CAMECA definition). Under these conditions, the ^18^O^–^ count per second (cps) was typically around 3.5 × 10^5^ cps. Surface contamination was removed by presputtering with a 2 nA primary beam over a 12 × 12 μm^2^ area for 2 min. Analytical data were then acquired from the central 3.5 × 3.5 μm^2^ region within a 5 × 5 μm^2^ raster, with ∼ 50% beam blanking.

Because this analytical setup involves a very large mass dispersion across the NanoSIMS secondary-ion optics (a mass span of 18, from ^1^H^–^ to ^18^O^–^), special care was required in detector tuning. In particular, ^18^O^–^ was measured on the farthest fixed detector position (R ≈ 680 mm), whereas ^1^H^–^ was collected at the near-end extreme position (R ≈ 160 mm). Accordingly, the deflection and focusing voltages for the corresponding detector-side optics were carefully optimized to ensure simultaneous transmission and stable secondary ions collection efficiency for both masses. An electron gun (E-Gun) was used for charge compensation. Because conductivity may vary among individual glass grains, we maintained a fixed electron-gun high voltage and instead adjusted the sample-stage high-voltage offset to maximize count rates and stabilize the ^18^O^–^ signal during analysis. Reference materials were analyzed together with unknowns to monitor instrumental stability and calibrate measured signals.^24^

Hydrogen isotope are expressed in delta notation, δD = [((D/H)_sample/(D/H)_SMOW) – 1] × 1000‰, where SMOW is the standard mean ocean water with a D/H ratio of 1.5576 × 10^–4^.^25^ A series of reference materials were used for calibrating the H_2_O content and hydrogen isotopes of the samples: Durango apatite (H_2_O = 0.0478 wt % and δD = −120 ± 5‰)^26^ and Kovdor apatite (H_2_O = 0.98 ± 0.07 wt % and δD = −66 ± 21‰),^27^ the MORB glass (H_2_O = 0.258 wt % and δD = −73 ± 2‰), and basaltic glass ALV-1833–11 (H_2_O = 1.2 wt %) (Table 1).^28^ Water contents were calibrated using the ^1^H^–^/^18^O^–^ signal ratio measured by NanoSIMS on the series reference materials.

**Table 1:** H_2_O Contents and δD Values of Reference Materials Used in This Work

	Raw measured ion ratios				Calibrated H_2_O content and δD values			
Filename	^2^D/^1^H	1σ	^1^H/^18^O	1σ	H_2_O (ppm)	1σ (ppm)	δD (‰)	1σ (‰)
KOV-1_1	1.88 × 10^–4^	4.21 × 10^–6^	5.50 × 10^–1^	8.20 × 10^–4^	9806	59.3	–61	22
KOV-1_2	1.81 × 10^–4^	3.90 × 10^–6^	5.55 × 10^–1^	7.66 × 10^–4^	9890	59.7	–94	22
KOV-1_3	1.89 × 10^–4^	3.94 × 10^–6^	5.39 × 10^–1^	7.38 × 10^–4^	9597	58.0	–53	21
KOV-1_4	1.89 × 10^–4^	4.08 × 10^–6^	5.61 × 10^–1^	8.20 × 10^–4^	10002	60.5	–56	22
DAP-1_1	1.72 × 10^–4^	1.90 × 10^–5^	2.52 × 10^–2^	4.54 × 10^–5^	449	2.7	–143	111
DAP-1_2	2.08 × 10^–4^	1.96 × 10^–5^	2.54 × 10^–2^	4.80 × 10^–5^	452	2.7	39	94
DAP-1_3	1.73 × 10^–4^	1.80 × 10^–5^	2.67 × 10^–2^	5.02 × 10^–5^	476	2.9	–134	104
MORB-1_1	1.57 × 10^–4^	7.97 × 10^–6^	1.56 × 10^–1^	1.64 × 10^–4^	2779	16.7	–218	51
MORB-1_2	1.52 × 10^–4^	7.88 × 10^–6^	1.57 × 10^–1^	1.84 × 10^–4^	2799	16.9	–239	52
MORB-1_3	1.81 × 10^–4^	8.76 × 10^–6^	1.55 × 10^–1^	1.77 × 10^–4^	2763	16.7	–94	48
1833–11–1_1	1.83 × 10^–4^	5.24 × 10^–6^	6.58 × 10^–1^	4.12 × 10^–4^	11716	70.4	–87	29
1833–11–1_2	1.68 × 10^–4^	4.38 × 10^–6^	6.62 × 10^–1^	5.51 × 10^–4^	11801	71.0	–158	26
1833–11–1_3	1.77 × 10^–4^	4.45 × 10^–6^	6.90 × 10^–1^	6.81 × 10^–4^	12288	74.0	–113	25
SCOL_1	3.51 × 10^–5^	3.51 × 10^–5^	1.46 × 10^–3^	1.79 × 10^–5^	26	0.2	-	-
SCOL_2	7.99 × 10^–5^	4.63 × 10^–5^	2.02 × 10^–3^	2.24 × 10^–5^	36	0.3	-	-

## Results and Discussion

### Fresh-Glass Purification and Analytical Targeting

Pure and fresh volcanic glass is a prerequisite for reliable in situ analysis of hydrogen isotopes and H_2_O contents. Previous bulk-shard protocols used prolonged HF abrasion to remove secondary minerals and altered surface material, and documented measurable weight loss before and after treatment, indicating that abrasion not only stripped alteration products but also dissolved part of the glass itself.^22^ Our HF-abrasion experiments show that brief HF treatment (40–50 s) serves as a limited surface-cleaning step rather than causing extensive bulk dissolution. Compared with untreated grains, HF-treated shards display cleaner surfaces, sharper shard and vesicle margins, and less fine-grained alteration residue, indicating effective removal of surficial alteration while minimizing unnecessary sample loss (Figure 1).

**Figure 1:**
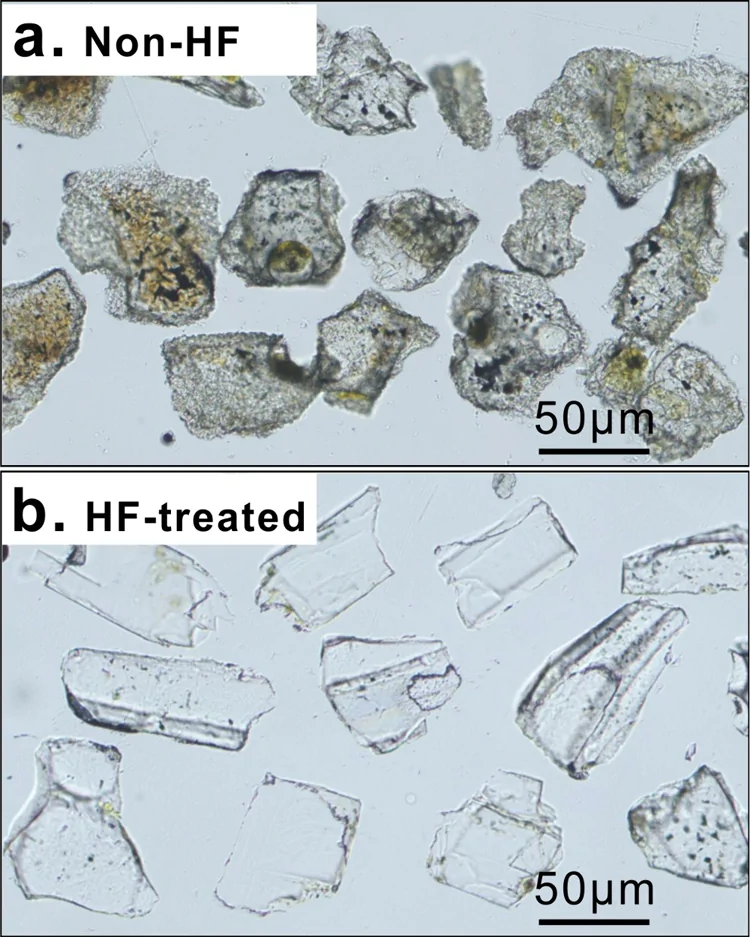
Reflected-light images of the volcanic glass. Non-HF treated grains (a) and HF-treated grains (b), showing that HF treatment effectively removes alteration products.

In addition, the NanoSIMS results show that presputtering with a high-current primary beam further helps avoid slightly altered areas and surrounding mounting materials, allowing precise determination of both hydrogen isotope and H_2_O contents in the fresh glass domains. Thus, although our protocol is less aggressive than previous bulk methods, it effectively removes surface alteration while preserving the primary hydration signal and is better suited to small, valuable, and texturally heterogeneous samples.

### Hydrogen Isotope and H_2_O Contents of Reference Materials

As shown in Figure 2 and Table 1, when the calibration is constrained to pass through the origin (i.e., assuming zero ^1^H^–^/^18^O^–^ at zero H_2_O), the derived slope is 0.5612 (R^2^ = 0.9998). To assess the hydrogen background, the ^1^H^–^/^18^O^–^ ratios were measured on SCOl. The ^1^H^–^/^18^O^–^ ratio measured on SCOl is around 1.5 × 10^–3^. According to the calibration described above, this ratio corresponds to a H_2_O content of ∼ 30 ppm, which is taken to represent the background contribution from the mount. For our samples, the background was corrected by subtracting the SCOl background. The resulting background level is higher than that reported in previous NanoSIMS studies.^29^ This difference likely reflects the analytical conditions used here. Because our samples have relatively high H_2_O contents and require limiting electron-multiplier aging, we used a lower primary beam current (350 pA) than the >1 nA currents commonly employed in earlier low-background studies. As a result, the measured background in this study is higher than in those previous reports. Compared with the H_2_O contents of the volcanic glass samples (>14,000 ppm), the mount background is very low relative to the sample signal and is unlikely to significantly affect the measurements.

**Figure 2:**
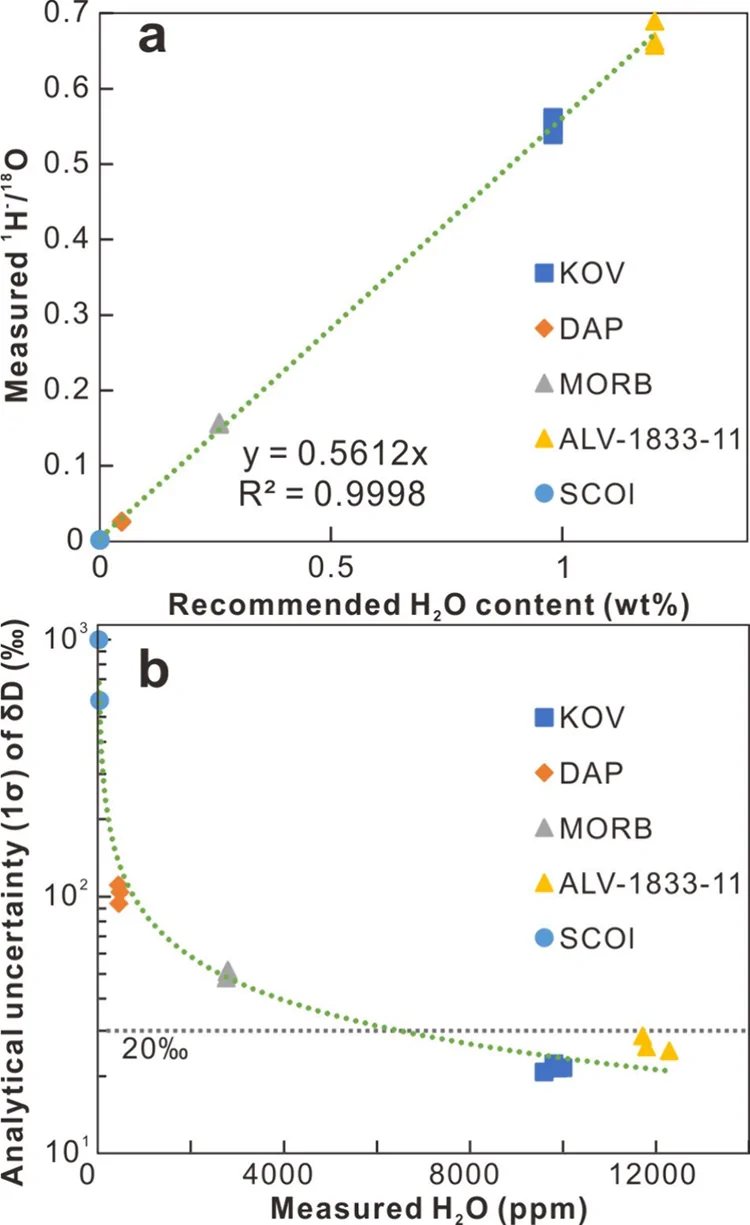
Calibration and analytical performance of reference materials. (a) Relationship between measured ^1^H^–^/^18^O^–^ ratios and recommended H_2_O content for KOV, ALV-1833–11, MORB, DAP and SCOl. (b) Relationship between measured H_2_O contents and of 1σ analytical uncertainty of δD. SCOl defines a hydrogen-background level equivalent to approximately 30 ppm of H_2_O. When the H_2_O content around 10,000 ppm (KOV), the analytical precision of the δD is approximately 20‰ (1σ).

The analytical precision of δD is primarily controlled by the H_2_O content of the standard materials. As shown in Figure 2b, δD uncertainty decreases systematically with increasing measured H_2_O content and follows a well-defined inverse power law relationship. In particular, when the H_2_O content around 10,000 ppm (KOV), the analytical precision of the H_2_O content and δD is around 15‰ and 20‰, respectively. The mean internal analytical precision of the ^2^H^–^/^1^H^–^ ratio is 20–22‰, consistent with the reproducibility of ∼ 20‰, suggesting that the measurement uncertainty is dominated chiefly by counting statistics (i.e., Poisson statistics). This agreement further indicates that no substantial additional uncertainty was introduced during sample preparation or instrumental analysis. Since the H_2_O contents of our volcanic glass samples are all higher than 14,000 ppm, comparable or better analytical precision (<20‰) can be expected.

### Hydrogen Isotope and H_2_O Contents of the Early Cretaceous Volcanic Glass and Their Implications

Based on this workflow, we report the first in situ hydrogen isotope and H_2_O content data for Early Cretaceous volcanic glass. Previous studies have reported magmatic H_2_O contents for Late Early Cretaceous volcanic rocks from the North China Craton, including the Yixian Formation, providing important constraints on volatile behavior during Early Cretaceous magmatism.^30,31^ The relatively high H_2_O contents is consistent with secondary hydration trends documented in experimental and natural volcanic glass studies, rather than with primary magmatic volatile contents.^32^ This distinction is important because freshly erupted volcanic glass commonly contains only minor residual magmatic water, whereas secondary hydration can substantially increase total H2O contents and lower δD values through interaction with meteoric water.

The H_2_O contents of the samples HBJ25–03 and HBJ25–05 are of range 14865–33766 ppm and 17664–23347 ppm, respectively (Table S1). Although the water contents of the samples show relatively large variability, their hydrogen isotope ratios are consistent, suggesting a common hydration-water source or a similar paleoenvironmental signal. The δD values for HBJ25–03 range from – 117‰ to – 33‰, and while those for HBJ25–05 range from – 106‰ to – 35‰ (Figure 3 and Table S1). The weighted mean δD values of HBJ25–03 and HBJ25–05 are – 68 ± 11‰ (2σ, 25 analyses) and – 63 ± 10‰ (2σ, 28 analyses), respectively. Compared with previous bulk TC/EA analyses of Cenozoic volcanic glass from North America,^8,22^ for which the best-case analytical uncertainty is typically around 3–4‰ under favorable conditions, the uncertainty of our in situ analyses (10–11‰) is higher, but remains sufficient to obtain robust δD estimates and to resolve major isotopic variability among individual grains.

**Figure 3:**
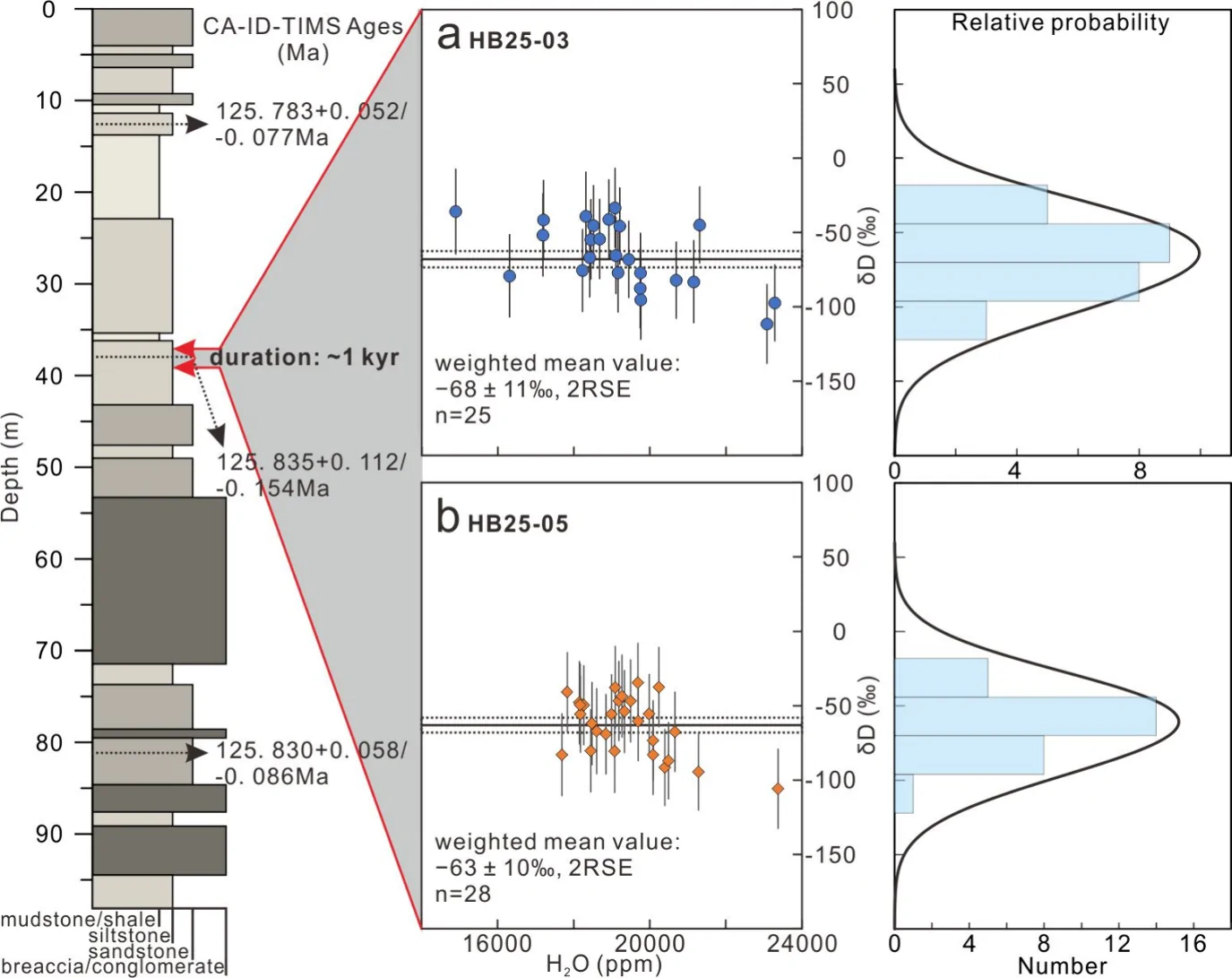
Stratigraphy, hydrogen isotope compositions, and H_2_O contents of the Early Cretaceous tuff samples from western Liaoning, NE China. Error bars in panels a and b are shown at the 1σ level.

Our approach analyzes individual grains at an ultralow sample mass (∼10^–9^ g), whereas TC/EA bulk measurements are generally performed on milligram-scale (∼10^–3^–10^–2^ g), homogenized glass separates. As a result, some of the larger scatter observed in previous bulk data sets may reflect interaliquot or intergrain heterogeneity in addition to analytical uncertainty, whereas in situ analysis can directly identify such microscale variability rather than averaging it out.^8^ This advantage is particularly important for older samples and scientific drilling core samples (Figure S1), for which the amount of fresh volcanic glass that can be separated is commonly far below the material requirements of bulk analysis. Therefore, this method provides an effective method of determining δD and H_2_O contents in ultralow-mass samples. This ultralow sample requirement is critical not only for rare low-volume core materials, but also for testing whether volcanic-glass δD can preserve meaningful paleoenvironmental signals at very high temporal resolution.

High-precision Chemical Abrasion-Isotope Dilution-Thermal Ion Mass Spectrometry (CA-ID-TIMS) U–Pb zircon geochronology together with anchored cyclostratigraphy constrains a mean sediment accumulation rate of ∼ 136.7 cm/kyr.^20^ The stratigraphic thickness between the two samples in this study is 124 cm, indicating that they represent an interval of only ∼ 1 kyr (Figure 3).^20^ Despite differing H_2_O contents, the two samples yield consistent δD values, implying that the isotopic composition of paleometeoric water remained stable, and by inference the associated topographic signal. This result further suggests that a common hydration-water source or a similar paleoenvironmental signal at millennial-scale stratigraphic resolution.^8,22^ Given previous studies indicating that postdepositional hydration of volcanic glass generally occurs over time scales of 1–10 kyr through diffusion and exchange with meteoric water,^8^ our results are consistent with the interpretation that volcanic-glass δD can record hydrologic and topographic information at approximately millennial scale resolution in the Early Cretaceous. More broadly, this approach provides an independent and promising line of evidence for quantitative paleoelevation reconstruction in this region, complementing existing carbonate clumped-isotope studies rather than relying on them alone.^33^ This opens a new window for ultrahigh-resolution paleoenvironmental reconstruction especially in ash rich successions with independent geochronological control.

## Conclusion

In summary, we establish a low-background, ultralow sample consumption in situ NanoSIMS method for measuring H_2_O contents and hydrogen isotope in hydrated volcanic glass. Applied to Early Cretaceous tuffs, it enables ∼ 10^–9^ g scale analysis of carefully screened fresh-glass microdomains and overcomes key limitations of conventional bulk methods for altered and sample-limited materials. This approach therefore extends volcanic-glass-based paleoenvironmental reconstruction into older and more poorly preserved strata, while also allowing substantially improved stratigraphic resolution.

## Supplementary Material



## Notes

Sampling photographs of samples in this study; H_2_O contents and δD values of volcanic glass samples (PDF)
