# Supplementary material for: Hydrogen Isotope and Water Content Analysis of Ultralow Volume Volcanic Glass
Source: ACS Meas Sci Au. 2026 Jul 4;6(4):923–8. doi: 10.1021/acsmeasuresciau.6c00092 (PMC13495710; doi:10.1021/acsmeasuresciau.6c00092)
Supplement: Supplementary file 1 [file tg6c00092_si_001.pdf]

Hydrogen isotope and water content analysis of ultralow volume volcanic glass

Zhiqiang Yu†, Jialong Hao†*

†Key Laboratory of Earth and Planetary Physics, Institute of Geology and Geophysics, Chinese Academy of Sciences, Beijing, 100029, China

*Correspondence: sean_hao@mail.iggcas.ac.cn (J-L. Hao);

Supplementary Figure.

Figure S1. Samples HB25-05 and HB25-03 from Huangbanjigou No. 1 core.

Table of Content.

Table S1. H_2O contents and δD values of volcanic glass samples.

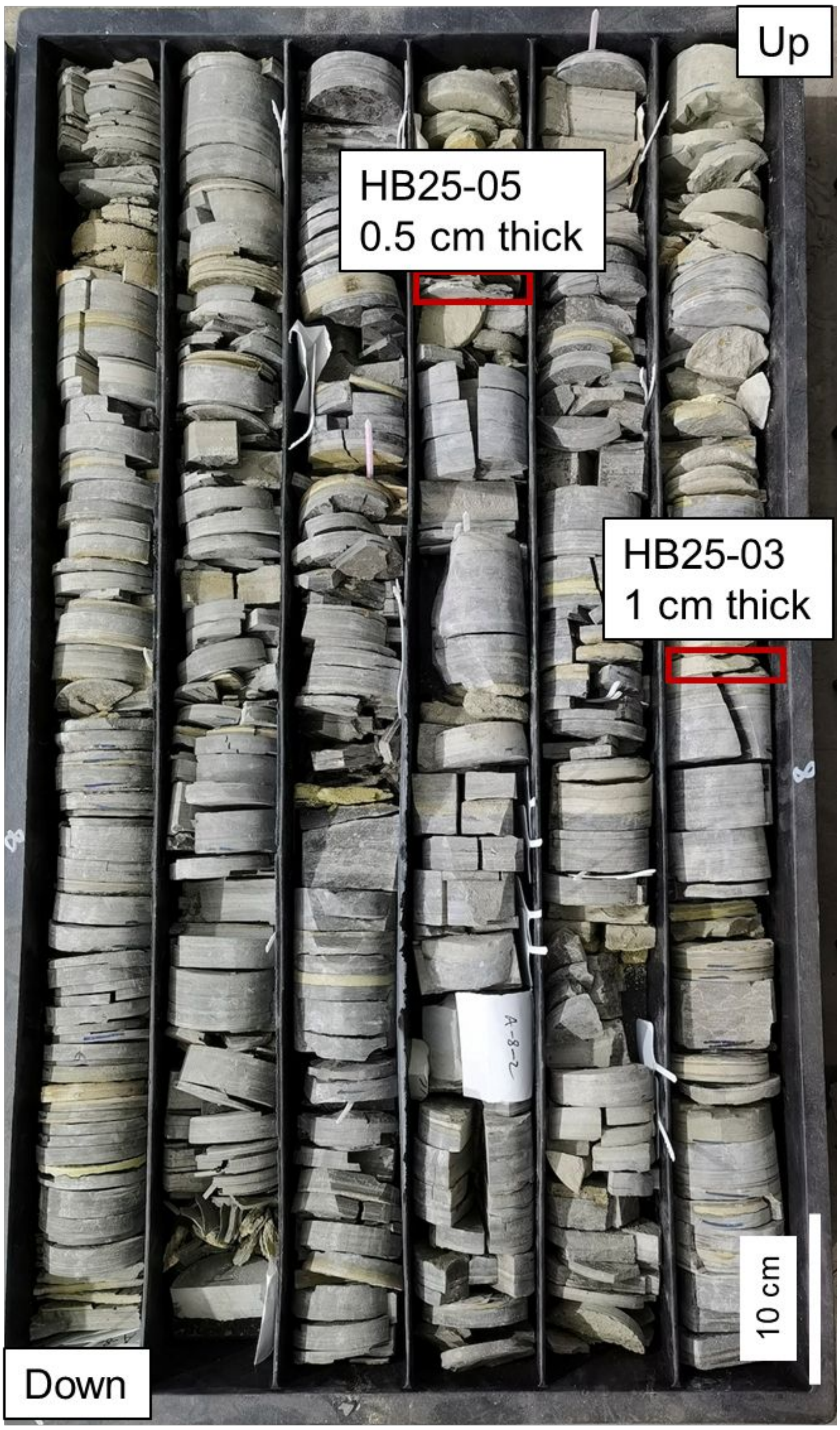

Fig. S1. Samples HB25-05 and HB25-03 from Huangbanjigou No. 1 core.

Supplementary Table S1. H_2O contents and δD values of volcanic glass samples.

Filename	$^{2}D/^{1}H$ Ratio	$^{2}D/^{1}H$ Err Mean (%)	$^{1}H/^{18}O$ Ratio	$^{1}H/^{18}O$ Err Mean (%)	$^{12}C/^{18}O$ Ratio	$^{12}C/^{18}O$ Err Mean (%)	H_2O (ppm)	1σ* (ppm)	δD (‰)	1σ# (‰)
HB25-3_1	1.92E-04	2.19E+00	1.03E+00	6.45E-02	2.31E-03	9.77E-01	18284	110	-39	30
HB25-3_2	1.78E-04	1.74E+00	1.30E+00	7.07E-02	4.83E-04	1.45E+00	23050	139	-112	27
HB25-3_3	1.93E-04	1.73E+00	1.07E+00	7.02E-02	2.60E-04	1.55E+00	19052	115	-33	27
HB25-3_4	1.90E-04	1.96E+00	9.65E-01	1.30E-01	5.77E-04	1.18E+00	17160	104	-52	28
HB25-3_5	1.91E-04	1.68E+00	1.08E+00	1.26E-01	3.23E-04	1.47E+00	19177	116	-46	26
HB25-3_6	1.81E-04	1.78E+00	1.11E+00	4.28E-02	1.11E-03	1.39E+00	19730	119	-95	27
HB25-3_7	1.92E-04	1.81E+00	9.65E-01	1.25E-01	2.47E-04	1.72E+00	17170	104	-42	27
HB25-3_8	1.84E-04	1.95E+00	9.15E-01	1.20E-01	1.55E-03	1.12E+00	16279	98	-79	28
HB25-3_9	1.77E-04	1.43E+00	1.90E+00	2.02E-01	5.65E-02	9.45E-01	33766	206	-117	25
HB25-3_10	1.86E-04	1.71E+00	1.09E+00	4.95E-02	2.47E-03	1.99E+00	19417	117	-68	26
HB25-3_11	1.85E-04	1.90E+00	1.02E+00	1.27E-01	2.05E-03	3.74E+00	18200	110	-76	28
HB25-3_12	1.91E-04	1.73E+00	1.04E+00	9.14E-02	6.62E-04	1.06E+00	18482	111	-45	27
HB25-3_13	1.89E-04	1.79E+00	1.04E+00	1.25E-01	9.06E-04	9.68E-01	18419	111	-55	27
HB25-3_14	1.85E-04	1.84E+00	1.08E+00	8.96E-02	6.08E-04	1.73E+00	19132	115	-77	27
HB25-3_15	1.87E-04	1.86E+00	1.03E+00	1.05E-01	2.17E-03	9.81E-01	18389	111	-67	27
HB25-3_16	1.89E-04	1.78E+00	1.05E+00	7.15E-02	5.61E-04	2.13E+00	18650	112	-54	27

HB25-3_17	1.85E-04	1.82E+00	1.11E+00	1.31E-01	1.25E-03	2.20E+00	19726	119	-77	27
HB25-3_18	1.83E-04	1.76E+00	1.11E+00	1.26E-01	7.10E-03	1.09E+00	19718	119	-88	27
HB25-3_19	1.87E-04	1.72E+00	1.07E+00	5.88E-02	1.13E-03	2.06E+00	19071	115	-66	26
HB25-3_20	1.83E-04	1.91E+00	1.19E+00	9.95E-02	1.33E-03	1.92E+00	21127	127	-83	28
HB25-3_21	1.92E-04	1.79E+00	1.06E+00	1.08E-01	4.06E-04	1.38E+00	18888	114	-41	27
HB25-3_22	1.81E-04	1.70E+00	1.31E+00	7.38E-02	1.53E-03	2.06E+00	23255	140	-97	26
HB25-3_23	1.93E-04	2.12E+00	8.36E-01	7.12E-02	2.07E-03	1.82E+00	14865	90	-36	29
HB25-3_24	1.84E-04	1.58E+00	1.16E+00	1.26E-01	7.64E-03	1.69E+00	20659	125	-82	26
HB25-3_25	1.91E-04	1.60E+00	1.20E+00	8.81E-02	3.51E-03	1.52E+00	21282	128	-45	26
HB25-5_1	1.89E-04	1.68E+00	1.02E+00	9.24E-02	1.60E-03	3.48E+00	18149	109	-56	26
HB25-5_2	1.93E-04	1.89E+00	1.07E+00	9.47E-02	7.00E-04	1.17E+00	19065	115	-38	28
HB25-5_3	1.91E-04	1.92E+00	1.02E+00	1.08E-01	3.25E-04	1.40E+00	18121	109	-48	28
HB25-5_4	1.89E-04	1.79E+00	1.12E+00	1.08E-01	1.06E-03	1.93E+00	19957	120	-56	27
HB25-5_5	1.88E-04	1.88E+00	1.04E+00	8.33E-02	2.16E-04	1.90E+00	18451	111	-62	28
HB25-5_6	1.84E-04	1.98E+00	1.07E+00	6.46E-02	1.74E-04	1.88E+00	19050	115	-81	28
HB25-5_7	1.82E-04	1.62E+00	1.14E+00	1.12E-01	1.04E-03	1.77E+00	20369	123	-92	26
HB25-5_8	1.92E-04	1.82E+00	1.00E+00	1.05E-01	4.21E-04	1.32E+00	17800	107	-41	27
HB25-5_9	1.79E-04	1.74E+00	1.31E+00	1.05E-01	8.16E-05	3.00E+00	23347	141	-106	27
HB25-5_10	1.83E-04	2.00E+00	9.93E-01	1.02E-01	4.21E-04	1.49E+00	17664	107	-83	28
HB25-5_11	1.84E-04	1.90E+00	1.04E+00	7.23E-02	7.15E-04	1.19E+00	18424	111	-80	28

HB25-5_12	1.89E-04	1.73E+00	1.07E+00	8.53E-02	1.68E-04	1.96E+00	18970	114	-56	27
HB25-5_13	1.90E-04	1.75E+00	1.03E+00	7.93E-02	8.02E-04	1.32E+00	18236	110	-50	27
HB25-5_14	1.93E-04	1.74E+00	1.14E+00	7.30E-02	2.41E-04	1.81E+00	20216	122	-38	27
HB25-5_15	1.87E-04	2.08E+00	1.04E+00	1.11E-01	7.45E-02	4.85E-01	18574	112	-67	29
HB25-5_16	1.90E-04	1.90E+00	1.02E+00	8.91E-02	4.19E-03	9.15E-01	18141	109	-49	28
HB25-5_17	1.89E-04	1.92E+00	1.09E+00	1.28E-01	3.10E-04	1.69E+00	19309	117	-54	28
HB25-5_18	1.88E-04	1.84E+00	1.11E+00	1.03E-01	1.71E-04	2.43E+00	19669	119	-60	27
HB25-5_19	1.87E-04	1.79E+00	1.16E+00	1.37E-01	7.40E-04	9.65E-01	20630	125	-68	27
HB25-5_20	1.83E-04	1.67E+00	1.15E+00	7.57E-02	1.88E-03	1.43E+00	20467	123	-87	26
HB25-5_21	1.81E-04	1.68E+00	1.19E+00	5.15E-02	3.48E-03	1.17E+00	21254	128	-95	26
HB25-5_22	1.91E-04	1.94E+00	1.09E+00	1.11E-01	2.35E-04	1.66E+00	19466	117	-47	28
HB25-5_23	1.93E-04	1.76E+00	1.11E+00	1.00E-01	7.39E-04	1.60E+00	19658	119	-35	27
HB25-5_24	1.91E-04	1.80E+00	1.08E+00	9.15E-02	1.75E-04	1.95E+00	19158	115	-47	27
HB25-5_25	1.86E-04	1.73E+00	1.06E+00	7.87E-02	4.70E-04	1.70E+00	18817	113	-69	27
HB25-5_26	1.83E-04	1.79E+00	1.13E+00	9.70E-02	7.82E-04	1.53E+00	20060	121	-83	27
HB25-5_27	1.91E-04	1.88E+00	1.08E+00	5.67E-02	4.61E-04	1.34E+00	19238	116	-44	28
HB25-5_28	1.85E-04	1.86E+00	1.13E+00	9.40E-02	5.09E-04	1.44E+00	20061	121	-73	27

Note: * The uncertainty of water content was estimated from two components: the internal uncertainty of the single-spot $^{1}H/^{18}O$ ratio measurement and the uncertainty of the calibration fit (15‰);

The uncertainty of δD was obtained by propagating the internal error of each analysis and the uncertainty associated with the reference materials (20‰).
